# Arabinogalactan Structures of Repetitive Serine-Hydroxyproline Glycomodule Expressed by Arabidopsis Cell Suspension Cultures

**DOI:** 10.3390/plants12051036

**Published:** 2023-02-24

**Authors:** Li Tan, Jianfeng Xu, Michael Held, Derek T. A. Lamport, Marcia Kieliszewski

**Affiliations:** 1 Complex Carbohydrate Research Center, University of Georgia, Athens, GA 30602, USA; 2 DOE Center for Plant and Microbial Complex Carbohydrates, University of Georgia, Athens, GA 30602, USA; 3 Arkansas Biosciences Institute, Arkansas State University, Jonesboro, AR 72401, USA; 4 Department of Chemistry and Biochemistry, Ohio University, Athens, OH 45701, USA; 5 School of Life Sciences, University of Sussex, Falmer, Brighton BN1 9QG, UK

**Keywords:** arabinogalactan-proteins, arabinogalactan polysaccharides, glycosylation, NMR

## Abstract

Arabinogalactan-proteins (AGPs) are members of the hydroxyproline-rich glycoprotein (HRGP) superfamily. They are heavily glycosylated with arabinogalactans, which are usually composed of a β-1,3-linked galactan backbone with 6-*O*-linked galactosyl, oligo-1,6-galactosyl, or 1,6-galactan side chains that are further decorated with arabinosyl, glucuronosyl, rhamnosyl, and/or fucosyl residues. Here, our work with Hyp-*O*-polysaccharides isolated from (Ser-Hyp)_32_-EGFP (enhanced green fluorescent protein) fusion glycoproteins overexpressed in transgenic Arabidopsis suspension culture is consistent with the common structural features of AGPs isolated from tobacco. In addition, this work confirms the presence of β-1,6-linkage on the galactan backbone identified previously in AGP fusion glycoproteins expressed in tobacco suspension culture. Furthermore, the AGPs expressed in Arabidopsis suspension culture lack terminal-rhamnosyl residues and have a much lower level of glucuronosylation compared with those expressed in tobacco suspension culture. These differences not only suggest the presence of distinct glycosyl transferases for AGP glycosylation in the two systems, but also indicate the existence of minimum AG structures for type II AG functional features.

## 1. Introduction

Arabinogalactan-proteins (AGPs) are diverse members of the hydroxyproline-rich glycoprotein (HRGP) superfamily and broadly implicated in many aspects of plant growth and development, ranging from cell proliferation to plant microbe interactions [1,2,3,4,5,6,7,8]. AGP family members include the hyperglycosylated classical AGPs; AGP chimeras that combine non-HRGP domains with AGP motifs; and AGP hybrids that mix AGP motifs with signature motifs of two other types of HRGPs, namely the extensins and/or proline-rich proteins [9]. Finally, there are many proteins that are not hydroxyproline-rich, yet contain a few short sequences [10], or ‘glycomodules’, that direct local glycosylation typical of AGPs [11].

AGPs are distinguished from other HRGP family members [12] by the presence of type II, type III (Mugwort) [13], or type I (Ginkgo) [14] arabinogalactan polysaccharides (AGs), which consist of a β-1,3-, β-1,6- or β-1,4-linked galactan backbone, respectively, as well as *O*-linked to hydroxyproline (Hyp) residues, although AGPs are traditionally referred to as HRGPs that are highly glycosylated with type II AGs. Carbohydrate occurring mainly as Hyp-*O*-linked AGs can account for as much as 95% of an AGP’s dry weight [1], elaborating most of the interactive surface. As such, the AG substituents are major determinants of AGP molecular function.

Type II AGs are branched heteropolysaccharides containing D-Gal*p*, L-Ara*f,* and often D-Glc*p*A and L-Rha*p* [15] attached to non-contiguous Hyp residues [16,17] that are often clustered in AGP glycomodule sequences X-Hyp-X-Hyp, where X is usually Ser or Ala, although lone Hyp residues can be targets as well [18]. In contrast, short *O*-linked arabinoside substituents decorating contiguous Hyp residues are minor components of AGPs but are the major glycans of the extensins and PRPs [18], which lack arabinogalactan polysaccharides. These differences in HRGP glycosylation give rise to different structures and chemistry, and thus different types of networks and biological functions. For example, extensins and PRPs form covalent wall networks through tyrosine crosslinking [19,20]*,* whereas classical AGPs occur primarily at the membrane–wall interface, intercellularly or in exudates [21,22], and participate in non-covalent and covalent networks through glycosidic bonds with pectin and xylan [23,24].

**Figure 1:**
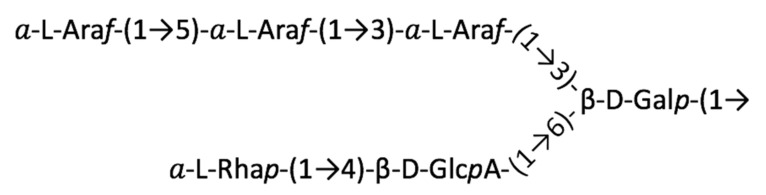
Scheme of the AGP side chain originated from the work of Defaye and Wong (1986) [26].

In our quest to relate structure to function, we previously used high-resolution one-dimensional (1D) and two-dimensional (2D) NMR techniques to elucidate the detailed glycan structure of four Hyp-*O*-AGs isolated from AGP fusion glycoproteins expressed in tobacco BY2 cells. Two Hyp-*O*-glycans, designated AHP1 (Ala-Hyp polysaccharide 1) and AHP2, were isolated from (Ala-Hyp)_51_-EGFP consisting of 51 repeats of an “Ala-Hyp” motif fused with enhanced green fluorescent protein (EGFP) [15,17], and two more were isolated from the fusion glycoprotein, human interferon α-2b (hIFNα-2b)-(Ser-Hyp)_20_ (20 repeats of a “Ser-Hyp” motif) [25]. All possessed common composition and linkage patterns, essentially differing only in the number and size of the side chains attached to the galactan mainchain, with the maximum side chain size being the six-residue unit proposed decades ago by Defaye and Wong (Figure 1) [26,27]:

With the discoveries of AGP-specific degrading enzymes in recent years, it is now possible to selectively remove specific side chain sugar residues or cleave the 1,3-galactan backbone [28,29,30]. Using this approach combined with gel electrophoresis and mass spectrometry analysis, Dupree and coworkers identified long β-1,6-galactan side chains from a variety of AGP or AG samples. This long side chain feature is corroborated by the identification of galactosyltransferases that can elongate the AG β-1,6-galactan side chain [31]. Thus, current data suggest that the AGP β-1,3-galactan backbone is elaborated with 6-*O*-linked galactosyl, oligo-1,6-galactosyl, or 1,6-galactan residues [32], which, at the periphery, have arabinosyl, glucuronosyl, rhamnosyl, and/or fucosyl residues.

AGP glycosylation is species- and tissue-dependent owing to the presence of genes encoding the various glycosyltransferases and the corresponding regulators that control their temporospatial expression [33]. However, we lack biochemical verifications of the different AGP glycosylations. This limits our understanding of the relationship between glycosylation and AGP function. Here, we used 1D and 2D NMR techniques together with glycosyl composition and glycosyl linkage analyses to characterize two Hyp-*O*-AGs isolated from the AGP fusion glycoprotein, (Ser-Hyp)_32_-EGFP, expressed in an Arabidopsis cell suspension culture [11]. Glycosylations of the two Hyp-*O*-AGs, designated Arabidopsis Ser-Hyp polysaccharides 1 and 2 (AtSHP-1 and AtSHP-2), were compared to those expressed in *Nicotiana*, which is only distantly related to Arabidopsis.

## 2. Results

### 2.1. Hyp-O-Polysaccharides from the Arabidopsis Fusion Glycoprotein (Ser-Hyp)_32_-EGFP

To analyze the glycosylation of Arabidopsis AGPs, a simple repetitive AGP glycomodule-EGFP fusion protein, (Ser-Hyp)_32_-EGFP, was expressed and purified from the culture media of transgenic Arabidopsis cells [11]. The fusion glycoprotein co-precipitated with Yariv reagent, demonstrating that the glycomodules were glycosylated with arabinogalactan polysaccharides. Glycosyl composition analysis of (Ser-Hyp)_32_-EGFP showed that it contained Ara, Gal, and GlcA, which are common residues in type II AGs. The fusion glycoproteins were hydrolyzed under mild base conditions to cleave the peptide backbone and release the Hyp-*O*-attached AG polysaccharides, which were fractionated on a size exclusion column that yielded a single peak containing Hyp and sugar residues [11]. The major fraction 17 and a later fraction 19, designated AtSPHP-1 and AtSPHP-2, respectively, were chosen for further detailed structural analyses.

### 2.2. Structure of AtSPHP-1

*AtSPHP-1 size and composition.* Our previous measurements of Hyp and monosaccharides of the fractionated Hyp-*O*-polysaccharides showed that they contained 18–27 glycosyl residues per Hyp [11], suggesting that these AGs ranged from 18 to 27 in degree of polymerization (DP). TMS glycosyl composition analysis yielded 45.1% Ara, 52.5% Gal, and 2.4% GlcA, while glycosyl linkage analysis produced linkages of 47.9% Ara*f*, 47.1% Gal*p*, and 5.0% t-Glc*p*A (Table 1). The increased mol% of GlcA in the glycosyl linkage analysis is consistent with the different sample hydrolysis conditions used in the two analyses (in 1 M methanolic HCl at 80 °C for 18 h for TMS glycosyl composition analysis vs. in 2 M TFA at 121 °C for 2 h for glycosyl linkage analysis), where the harsh hydrolysis obviously hydrolyzed more GlcA for derivatization and subsequent detection. It also suggests that AtSPHP-1 is composed of Ara/Gal/GlcA sugar residue in a ratio of 10:10:1. In summary, our analyses indicate AtSPHP-1 was a 21-residue glycan *O*-linked to Hyp.

**Figure 2:**
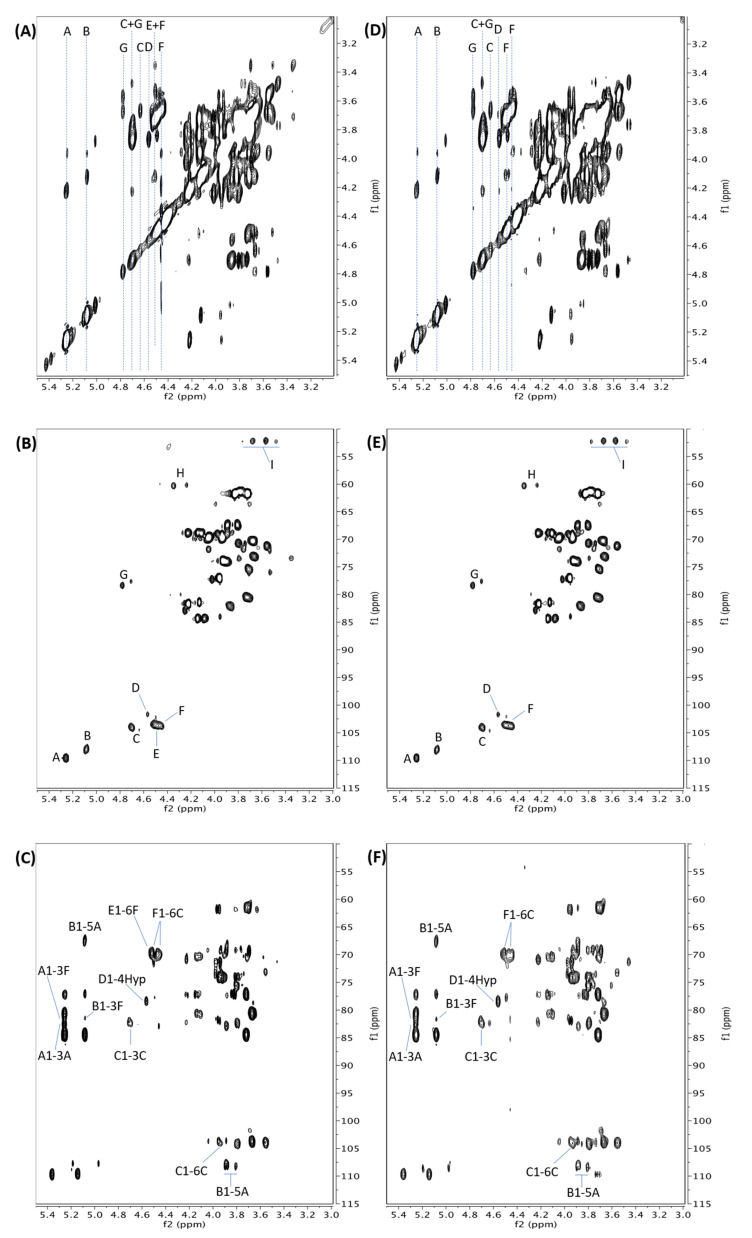
NMR spectra of AtSPHP-1 and -2 collected at 55 °C. (**A**–**C**) Spectra of AtSPHP-1 and (**D**–**F**) spectra of AtSPHP-2. (**A**,**D**): TOCSY spectra; (**B**,**E**): HSQC spectra; (**C**,**F**): HMBC spectra. Inside each spectrum, A and B represent α-L-Ara*f*; C as β-D-Gal*p* on galactan backbone; D as β-D-Gal*p* linked to Hyp; E as signals of β-D-Glc*p*A; F as signals side chain β-D-Gal*p*; and G, H, and I as the signals Hyp at positions 4, 2, and 5, respectively. In (**C**,**F**), each label represents a glycosidic linkage between two glycosyl residues, i.e., A1-3A represents HMBC correlation corresponding to Ara*f*-(1→3)-Ara*f* linkage.

A set of NMR spectra of AtSPHP-1, including Correlated Spectroscopy (COSY), Total Correlation Spectroscopy (TOCSY), Heteronuclear Single Quantum Coherence (HSQC), and Heteronuclear Multiple Bond Correlation (HMBC) spectra, were collected and analyzed to elucidate the chemical structure. ^1^H signals of individual glycosyl or Hyp residue were identified in the COSY and TOCSY spectra (Figure 2A), while the corresponding ^13^C signals were found in the HSQC spectrum (Figure 2B). We confirmed the ^1^H/^13^C assignments from the spectra and anomeric configurations of the saccharide residues (Table 2). The linkages between these residues were established based on correlations in the HMBC (Figure 2C).

*L-Hyp and allo-Hyp.* The presence of two sets of characteristic cross peaks in the HSQC spectrum (cross peaks G, H, and I in Figure 2B) indicated that the Hyp residues were a mixture of L-Hyp and allo-Hyp isomers formed during base hydrolysis of the polypeptide backbone [15,34].

*Side chain characterization.* Based on glycosyl composition data, AtSPHP-1 was composed of 10 Ara residues. The glycosyl linkage result further showed that the 10 Ara included four t-Ara*f*, three 5-Ara*f*, and three 3-Ara*f* residues. This is consistent with a 6:4 integral ratio of anomeric signal A to B in Figure 2B, which corresponded to six 3-Ara*f* and 5-Ara*f* and 4 t-Ara*f*, respectively. The presence of three 5-Ara*f* in the molecule was also supported by a 3:7 integral ratio of Ara*f* C/H-5 signals between 67.3/3.81, 3.88 ppm (5-Ara*f* C/H-5) and 62.0/3.82, 3.71 ppm (t- and 3-Ara*f* C/H-5). The HMBC correlations B1-5A (Ara*f* H-1 at 5.09 ppm to Ara*f* C-5 at 67.3 ppm) and B1-3F (Ara*f* H-1 at 5.09 ppm to Gal*p* C-3 at 80.9 ppm) showed that some t-Ara*f* were 1→5 linked to 5-Ara*f* and the other(s) were/was 1→3 linked to side chain Gal*p* residue(s) (Gal_sc_) (Figure 2C). In addition, HMBC correlations A1-3F (Ara*f* H-1 at 5.24 ppm to Gal*p* C-3 at 80.9 ppm) and A1-3A (Ara*f* H-1 at 5.24 ppm to Ara*f* C-3 at 83.0 ppm) demonstrated that some Ara*f* were 1→3 linked to 3-Ara*f* and the others were 1→3 linked to Gal_sc_ residues. Thus, our results suggest that the Ara*f* residues formed four structural units that substituted Gal_sc_ residues, among which three units were t-Ara*f*-(1→5)-Ara*f*-(1→3)-Ara*f*-(1→3)-Gal_sc_ and one t-Ara*f*-(1→3)-Gal_sc_. The number of Ara*f* substitutions also advocates that there were four Gal_sc_ residues in this polysaccharide and, correspondingly, six Gal*p* residues on the galactan backbone (Gal_bb_).

**Figure 3:**
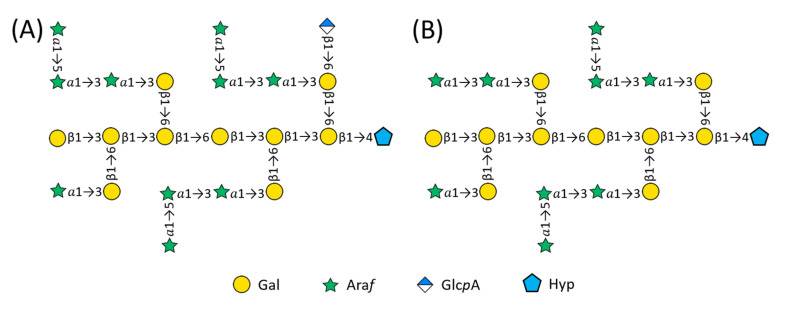
Proposed structures of AtSPHP-1 and -2. (**A**) AtSPHP-1 is composed of β-Gal*p*, α-Ara*f*, β-Glc*p*A, and Hyp in a ratio of 10:10:1:1. (**B**) AtSPHP-2 contains β-Gal*p*, α-Ara*f*, and Hyp in a ratio of 10:9:1. The 3,6-galactan structure is conserved based on the analyses. However, the locations of single Ara*f*, di-Ara*f*, tri-Ara*f*, and Glc*p*A on the side chain Gal*p* may not be exactly as shown above, i.e., the lone Glc*p*A residue can be attached to 6-*O* of any side chain Gal*p* of AtSPHP-1.

Furthermore, the correlation E1-6F (Glc*p*A H-1 at 4.51 ppm to side chain Gal*p* C-6 at 70.0 ppm) confirmed the lone Glc*p*A residue was 1→6 linked to a Gal_sc_ residue. However, we could not determine which of the four Gal_sc_ was substituted and we chose one in Figure 3A. Likewise, we assigned the α-L-Ara*f*-(1→3) side chain to another Gal_sc_, although any one of the four Gal_sc_ residue were candidates. Therefore, the Gal_sc_ included three 3-Gal*p* and one 3,6-Gal*p* that were 1→6 linked to backbone, and the galactan backbone consisted of one t-Gal*p*, one 6-Gal*p*, and four 3,6-Gal*p* residues.

*The Galactan Backbone of AtSPHP-1.* Cross peak D1-4Hyp (Gal*p* H-1 at 4.56 ppm to Hyp C-4 at 78.3 ppm) in the HMBC spectrum (Figure 2C) identified the backbone β-D-Gal*p* linkage to *O*-4 of Hyp (Table 2: Gal residue D, Hyp Set 1). The correlation C1-3C (Gal*p* H-1 at ~4.7 ppm to Gal*p* C-3 at 82.8 ppm) identified backbone Gal*p* residues (Gal_bb_) that were β-1→3 linked, while cross peak C1-6C (Gal*p* C-1 at 104.2 ppm to Gal*p* H-6 at 3.92 ppm) corroborated a β-1→6 linkage in the galactan backbone. Thus, with the presence of one t-Gal*p*, one 6-Galp, and four 3,6-Galp residues on the backbone and in light of earlier work suggesting a repetitive AG structure [25,35,36,37], we concluded the Gal backbone and Hyp residue were linked as follows: β-D-Gal*p*-(1→3)-β-D-Gal*p*-(1→3)-β-D-Gal*p*-(1→6)-β-D-Gal*p*-(1→3)-β-D-Gal*p*-(1→3)-β-D-Gal*p*-(1→4)-Hyp. As such, the backbone of AtSPHP-1 was identical to that of the 14-residue tobacco AHP-2 glycan characterized earlier [34] and its structure is proposed as shown in Figure 3A.

### 2.3. Structure of AtSPHP-2

*AtSPHP-2 size and composition*. As a later fraction from the size exclusion column, AtSPHP-2 was smaller than AtSPHP-1, differing in the extent of side chain elaboration. TMS glycosyl analysis of AtSPHP-2 indicated the Gal/Ara molar ratio was 10:9 (Table 1). Considering our previous results showing the AG sizes were 18–27 glycosyl residues per Hyp [11], the structural formula of AtSPHP-2 was Hyp_1_Gal_10_Ara_9_.

*The side chains units*. Glycosyl linkage analysis of AtSPHP-2 showed that it was composed of t-Ara*f*, 3-Ara*f*, and 5-Ara*f* in a ratio of 2.27:1.54:1, suggesting it consisted of four t-Ara*f*, three 3-Ara*f*, and two 5-Ara*f* residues. The presence of four t-Ara*f* moieties was supported by a 5:4 integral ratio of anomeric signal A to B in Figure 2E, showing that the sample contained five residues of 3-Ara*f* and 5-Ara*f* and four moieties of t-Ara*f*. The HMBC cross peaks B1-5A (Ara*f* H-1 at 5.09 ppm to Ara*f* C-5 at 67.9 ppm) and B1-3F (Ara*f* H-1 at 5.09 ppm to Gal*p* C-3 at 80.5 ppm) corroborated that some t-Ara*f* were 1→5 linked to 5-Ara*f* and the others were 1→3 linked to Gal_sc_ (Figure 2F) (Table 3). HMBC correlations A1-3F (Ara*f* H-1 at 5.25 ppm to Gal*p* C-3 at 80.5 ppm) and A1-3A (Ara*f* H-1 at 5.25 ppm to Ara*f* C-3 at 82.6 ppm) showed that some Ara*f* were 1→3 linked to 3-Ara*f* and the others were 1→3 linked to Gal_sc_ residues. Therefore, we concluded that there were four substitutions on *O*-3 of four Gal_sc_ residues, including two units of t-Ara*f*-(1→5)-Ara*f*-(1→3)-Ara*f*-(1→3)-Gal_sc_, one unit of t-Ara*f*-(1→3)-Ara*f*-(1→3)-Gal_sc_, and one unit of t-Ara*f*-(1→3)-Gal_sc_ (Figure 3).

*The galactan backbone of AtSPHP-2*. The presence of two sets of characteristic cross peaks in the HSQC spectrum (cross peaks G, H, and I in Figure 2E) indicated the Hyp residues were a mixture of L-Hyp and allo-Hyp isomers as AtSPHP-1 (Table 3) [15,34]. As with AtSPHP-1, cross peaks in the HMBC spectrum of AtSPHP-2 (Figure 2F) established the sequence of the galactan backbone, which was identical to that of AtSPHP-1 and AHP-2 [34].

## 3. Discussion

### AtSPHP-1 and AtSPHP-2 Are Variations on a Conserved Theme

As previously reported, arabinogalactan polysaccharides isolated from endogenous AGPs can contain hundreds of glycosyl residues [11,37]. We also observed that the sizes of Hyp-*O*-polysaccharides yielded from native AGPs purified from culture media of Arabidopsis suspension culture ranged from 65 to 142 glycosyl residues per Hyp [11]. However, Hyp-*O*-polysaccharides generated from the overexpression of AGP glycomodule- or AGP-recombinant proteins fused with EGFP or hIFNα-2b, either produced by transgenic tobacco or Arabidopsis suspension culture, were much smaller, with an average size of around 15 to 22 sugar residues. These Hyp-*O*-polysaccharides include AHP-1 and -2 from (Ala-Hyp)_51_-EGFP, Hyp-polysaccharide-1 and -2 from hIFNα-2b-(Ser-Hyp)_20_, and Hyp-glycan from rhGH-(Ser-Hyp)_10_, all overexpressed by tobacco cells [11,15,38], as well as AtSPHP-1 and -2 from this work. The smaller AG size is most likely due to the overexpression of the corresponding fusion glycoproteins/glycomodules and the inability of the glycosylation enzymes to completely glycosylate all of the abundant polypeptides expressed by the translational machinery driven by the 35S CaMV promoter. Therefore, the limited glycosylation enzymes and sugar donors resulted in short polysaccharides attached on the polypeptides. Future co-expression of AGP-specific glycosyltransferases and nucleotide sugar interconverting enzymes with (Ser-Hyp)_32_-EGFP may produce fusion glycoproteins with longer arabinogalactan polysaccharides.

Recent work demonstrated the existence of long β-1,6-galactan side chain 1→6-linked to the AGP galactan backbone [29,30]. The results showed that up to 30% of AG side chains were long β-1,6-galactans decorated with Ara, GlcA, and other minor glycosyl residues. However, it is hard to identify the long β-1,6-galactan side chain in AtSPHP-1 and -2, mainly because of the short average length of the AGs. Furthermore, NMR methods that can only provide an average picture of all of the polysaccharides in the sample make it hard to distinguish possible minor long β-1,6-galactans from the whole population.

Our HMBC spectra clearly show the existence of β-1,6-linkage on the AG galactan backbone. The simple patterns of the HSQC spectra suggest that tobacco AHP-1 and AHP-2 and Arabidopsis AtSPHP-1 and AtSPHP-2 share a repeating structural motif. They have a galactan backbone comprised of two β-1→3 trigalactosyl blocks connected by a β-1→6 linkage, although one block of AHP-1 is truncated, lacking a Gal at the non-reducing end of the backbone. The kink structural feature of AG galactan backbone is reminiscent of the backbone repeats of mixed-linkage glucan (MLG) from Poales [39]. The biosynthetic mechanism of MLG may have some relevance for AGP galactan backbone assembly and warrants future investigation. In addition, side chains, when present on the trigalactosyl blocks, initiate with Gal_sc_ residues β-1→6 linked to the first and second Gal_bb_ residues in the trigalactosyl blocks, numbering from the reducing end. When present, α-linked arabinosyl chains ranging in size from one to three residues are α-1→3-linked to the Gal_sc_ residues, while β-linked Glc*p*A occupies the *O*-6 position. Thus, such trigalactosyl units with the decorated carbohydrates may serve as the building blocks for large AG polysaccharides. This is supported by the fact that the short AtSPHP-1 and -2 and the large Hyp-*O*-AGs of native AGPs isolated from Arabidopsis suspension culture media are composed of Ara, Gal, and GlcA in similar molar ratios [11].

Our recent work on an RG-I-AGP complex released from cell walls of Arabidopsis suspension cultured cells by endopolygalacturonase suggests that the terminal Rha of AGPs may serve as the attachment site of RG-I [24]. Notably, the Arabidopsis AGs lacked the terminal α-L-rhamopyranosyl residues 4-linked to Glc*p*A in tobacco AHP-1 and AHP-2. Is the lack of Rha due to the lack of corresponding rhamnosyl transferase activity in the suspension cultured Arabidopsis cells? Where does the AGP Rha of RG-I-AGP come from? This apparent contradiction is indeed related to how the RG-I-AGP is synthesized. One possibility is that the Rha addition in Arabidopsis is regulated and only occurs when the assembly of RG-I-AGP complexes is needed, which requires a precisely controlled expression of AGP-specific rhamnosyl transferases. Another possibility is that the intermolecular Rha between the AGP and RG-I originates from the Rha at the reducing end of an RG-I glycan that is transferred to the terminal Glc*p*A of an AGP by possible pectin transglycanases. Nevertheless, it is worth confirming whether any genes encoding AGP-specific rhamnosyl transferases and the corresponding enzyme activity are present in these cells when sequences of such rhamnosyl transferases become available.

Another observation is that AtSPHP-1 and -2 from Arabidopsis cell culture underwent less gluconosylation than AHP-1 and -2 from tobacco cell culture. A similar trend was also observed by comparing GlcA amounts in EGFP-LeAGP-1 overexpressed by Arabidopsis and tobacco cells, which showed a significantly lower amount of GlcA in the major Hyp-*O*-polysaccharide fractions prepared from Arabidopsis EGFP-LeAGP-1 [11]. However, although a minor component, the conserved presence of GlcA in bifurcated calcium-binding [40] AG side chains might be directly related to their global biological significance, as demonstrated recently [8,32], which suggests binding and releasing apoplastic calcium is a function of AGPs.

A conserved backbone structure with different side chain decoration seems the major theme of AGP glycosylation in different plant species and tissues. Indeed, co-precipitation with β-Gal Yariv reagent, a common feature of AGPs, only requires a conserved β-1,3-galactan with a degree of polymerization (DP) greater than five [41]. Here, the average galactan backbone length of Arabidopsis (Ser-Hyp)_32_-EGFP able to co-precipitate with Yariv reagent is six, which is above the minimal requirement of five and close to seven, with the DP leading to more efficient Yariv precipitation. On the other hand, the difference in side chain modifications not only suggests the presence of distinct glycosyl transferases and possibly glycosyl hydrolases for AGP glycosylation and maturation between the two species, but also indicate the existence of minimum AG structures required for their *in vivo* functions [41]. Future identification and classification of AGP glycosyl transferases in different plant species and tissues will help us understand the evolution and divergency of AGP glycosylation, and thus the functions of AGP glycosylation in plants.

## 4. Materials and Methods

### 4.1. Isolation of (Ser-Hyp)_32_-EGFP from Arabidopsis Suspension Cultured Cells

Arabidopsis cells were transformed with the *(Ser-Pro)_32_-EGFP* gene cassette and cell lines expressing the fusion glycoprotein selected and cultured as described earlier [11,18]. (Ser-Hyp)_32_-EGFP was isolated from culture media by a combination of hydrophobic interaction chromatography and reverse-phase chromatography, as described earlier [11,18]. In brief, culture media of the transformed Arabidopsis cells were filtered and concentrated via rotary evaporation at room temperature. Solid NaCl was added to the concentrated media to a final concentration of 2 M, which was loaded on a hydrophobic interaction column (Phenyl Sepharose 6 Fast Flow, 16 × 700 mm, Amersham-Pharmacia Biotech, Amersham, UK). The bound materials were eluted from the column with distilled water and dialyzed against distilled water using 3.5 kDa MWCO dialysis tubing (Spectrum Chemical, New Brunswick, NJ, USA), concentrated via lyophilization, and separated on a semipreparative reverse-phase column (10 μm, PRP-1, 7 × 305 mm, Hamilton) at a flow rate of 1 mL/min using a gradient of 100% (*v*/*v*) buffer A (0.1% [*v*/*v*] aqueous trifluoroacetic acid) increasing to 70% (*v*/*v*) buffer B (80% [*v*/*v*] acetonitrile in 0.1% aqueous trifluoroacetic acid) for 105 min. The eluent of separation was monitored at 220 nm on a 1050 HPLC system (Hewlett-Packard, Novi, MI, USA). The (Ser-Hyp)_32_-EGFP eluted in about 50% (*v*/*v*) buffer B.

### 4.2. Co-Precipitation with Yariv Reagent

We assayed the reactivity of reverse-phase chromatography isolated fractions with β-galactosyl Yariv reagent using tobacco AGPs as a standard, as described earlier [17,18]. In brief, one hundred micrograms of (Ser-Hyp)_32_-EGFP was dissolved in 300 μL of dd H_2_O, followed by the addition of 300 μL of (β-D-galactosyl)_3_-Yariv reagent at a concentration of 1 mg/mL in 2% [*w*/*v*] NaCl aqueous solution. The mixture was incubated at room temperature for 1 h, followed by centrifuging at 6000× *g* to collect the precipitates. The precipitates were washed with 2% NaCl aqueous solution and dissolved in 0.1 M NaOH. The absorbance of the solution was measured at 420 nm and compared to the AGP standard.

### 4.3. Isolation of Hyp-O-Glycans

The (Ser-Hyp)_32_-EGFP (40 mg) were separately dissolved in 2 mL of 0.44 N NaOH (aqueous) and heated at 108 °C for 20 h. The hydrolysate was cooled on ice and titrated to pH 7.8 with 1 M HCl. The neutralized solution was then freeze-dried.

The hydrolysate was dissolved in 1 mL of distilled water and fractionated on an analytical Superdex peptide column (Amersham Biosciences, Piskataway, NJ, USA) eluted with 20% acetonitrile (aqueous) at a flow rate of 0.3 mL/min [11]. Fractions (0.6 mL per fraction) were assayed for Hyp and neutral sugar. High-molecular-weight fractions containing Hyp and sugar from the (Ser-Hyp)_32_-EGFP hydrolysate were selected and then re-fractionated on the Superdex peptide column before further analyses.

### 4.4. Sugar Analyses and Hyp Assay

Neutral sugars were analyzed as alditol acetate derivatives by gas chromatography (GC) using a 6-foot × 2 mm PEG succinate 224 column programmed from 130° to 180° at 4 °C/min [11,17]. Data were captured by Hewlett-Packard Chem station software. One hundred micrograms of glycoprotein was used for each analysis. We assayed the uronic acid content of 70 μg of each sample via the specific colorimetric assay based on the reaction with m-hydroxydiphenyl [17]. Glucuronic acid was the standard. Glycosyl compositions were also analyzed by GC/mass spectrometry (GC/MS) of trimethylsilyl derivatives of methyl glycosides, as previously described [42]. Glycosyl linkage analysis was performed by combined GC/MS of the partially methylated alditol acetate (PMAA) derivatives produced from the sample, using a procedure that was slightly modified from a previously described method [43], in which uronic acid components of each sample were methylated and reduced using lithium aluminum deuteride (LiAlD4) prior to PMAA derivatization.

Hydroxyproline was assayed colorimetrically using the Kivirikko’s method [17], which involves alkaline hypobromite oxidation and subsequent coupling with acidic Ehrlich’s reagent and monitoring at 560 nm.

### 4.5. NMR Analyses

All NMR spectra were collected at the Campus Chemical Instrument Center, Ohio State University. Two milligrams of each Hyp-*O*-glycan isolated from (Ser-Hyp)_32_-EGFP was dissolved in 0.7 mL of D_2_O. Because the water signal occurred in the anomeric region of the spectrum, 1-D ^1^H NMR spectra and 2-D TOCSY, COSY, HSQC, and HMBC spectra were recorded at 55 °C on a Bruker 800 MHz DRX spectrometer outfitted with a cryoprobe [15,34]. HSQC experiments were carried out with spectral width of 8 kHz for ^1^H and 30 kHz for ^13^C, respectively. ^1^J_CH_ was set to 150 Hz with a relaxation delay of 1.5 s. For HMBC spectra, the dataset was acquired at 4 × 1 K data points with multibounds J_CH_ at 8 Hz. DQCOSY spectra were collected with a spectral width of 8 kHz in both dimensions with a relaxation delay of 2 s. For TOCSY experiments, the mixing time was set at 90 ms. All data were processed using NMRPipe and analyzed using nmrview [15,34].

## 5. Conclusions

Compared with the AG structures of AGP glycomodules expressed by tobacco suspension cultured cells, the AGP glycomodules produced by Arabidopsis suspension cultures adopt a conserved theme on the AG backbone, while the side chain has different patterns of glycosyl additions. Specifically, Arabidopsis AGPs lack terminal rhamnose decoration and have a much lower level of glucuronosylation.

## Figures and Tables

**Table 1:** Glycosyl composition and glycosyl linkages of AtSPHP-1 and -2. All values are mol %.

AtSPHP-1				AtSPHP-2			
Glycosyl Residues	Mol%	Glycosyl Linkages	Mol%	Glycosyl Residues	Mol%	Glycosyl Linkages	Mol%
Ara	45.1	t-Ara*f*	18.9	Ara	45.8	t-Ara*f*	21.8
		3-Ara*f*	13.9			3-Ara*f*	14.8
		5-Ara*f*	15.1			5-Ara*f*	9.6
Gal	52.5	t-Gal*p*	4.6	Gal	54.2	t-Gal*p*	4.9
		3-Gal*p*	13.9			3-Gal*p*	21.2
		6-Gal*p*	4.5			6-Gal*p*	5.5
		3,6-Gal*p*	24.1			3,6-Gal*p*	22.2
GlcA	2.4	t-Glc*p*A	5.0	GlcA	trace	t-Glc*p*A	trace

**Table 2:** Summary of ^13^C and ^1^H chemical shifts for AtSPHP-1 assigned from Figure 2. Gal_bb_: Gal*p* on galactan backbone; Gal_sc_: Gal*p* on AG side chain.

Sugar/Hyp Residue		C-1/H-1	C-2/H2	C-3/H-3	C-4/H-4	C-5/H-5	C-6/H-6
B	t-α-Ara*f*	108.0/5.09	81.9/4.21	77.5/4.00	85.0/4.13	62.0/3.82, 3.71	
A	5-α-Ara*f*	109.5/5.24	81.9/4.21	77.0/3.96	85.0/4.13	67.3/3.81, 3.88	
A	3-α-Ara*f*	109.5/5.24	81.8/4.11	83.0/4.25	85.0/4.09	62.0/3.82, 3.71	
E	t-β-Glc*p*A	103.8/4.51	73.7/3.34	76.2/3.74	71.0/3.54	76.0/3.52	
F	3,6-β-Gal_sc_	104.0/4.46	70.2/3.66	80.9/3.72	69.1/4.10	74.2/3.88	70.0/4.03, 3.92
F	3-β-Gal_sc_	104.0/4.46	70.2/3.66	80.9/3.72	69.1/4.10	75.8/3.71	61.4/3.76
F	3-β-Gal_sc_	104.0/4.46	70.2/3.66	80.9/3.70	69.1/4.10	75.8/3.71	61.4/3.76
D	3,6-β-Gal_bb_	102.0/4.56	70.2/3.70	82.8/3.82	69.2/4.21	73.9/3.79	70.0/4.03, 3.92
C	3,6-β-Gal_bb_	104.6/4.68	70.2/3.78	82.8/3.84	69.2/4.21	73.9/3.79	70.0/4.03, 3.92
C	6-β-Gal_bb_	104.3/4.63	72.4/3.62	73.1/3.67	69.0/3.96	73.9/3.79	70.0/4.03, 3.92
C	3,6-β-Gal_bb_	104.3/4.68	70.2/3.78	82.8/3.86	69.2/4.21	73.9/3.79	70.0/4.03, 3.92
C	3,6-β-Gal_bb_	104.3/4.70	70.2/3.79	82.8/3.88	69.2/4.21	73.9/3.79	70.0/4.03, 3.92
C	t-β-Gal_bb_	104.3/4.68	72.4/3.72	73.1/3.66	69.0/3.88	75.8/3.70	61.4/3.76
Hyp	set 1		60.2/4.32	35.8/2.63,2.20	78.3/4.78	52.3/3.68, 3.56	
	set 2		60.0/4.22	35.2/2.55,2.45	77.9/4.69	52.3/3.78,3.48	

**Table 3:** Summary of ^13^C and ^1^H chemical shifts for AtSPHP-2 assigned from Figure 2. Gal_bb_: Gal*p* on galactan backbone; Gal_sc_: Gal*p* on AG side chain.

Sugar/Hyp Residue		C-1/H-1	C-2/H2	C-3/H-3	C-4/H-4	C-5/H-5	C-6/H-6
B	t-α-Ara*f*	108.0/5.09	81.9/4.21	77.3/4.01	84.3/4.13	61.8/3.82, 3.71	
A	5-α-Ara*f*	109.5/5.25	81.9/4.21	77.0/3.95	84.3/4.13	67.9/3.79, 3.88	
A	3-α-Ara*f*	109.5/5.25	81.4/4.11	82.6/4.24	84.3/4.08	61.8/3.82, 3.71	
F	3-β-Gal_sc_	104.0/4.45	70.2/3.66	80.5/3.73	69.1/4.10	75.1/3.72	61.2/3.78
F	3-β-Gal_sc_	104.0/4.51	71.3/3.71	80.5/3.73	69.1/4.10	75.1/3.72	61.2/3.78
D	3,6-β-Gal_bb_	102.0/4.56	70.2/3.70	83.0/3.86	69.0/4.21	73.5/3.79	69.9/4.04, 3.93
C	3,6-β-Gal_bb_	104.6/4.63	70.2/3.68	83.0/3.88	69.0/4.21	73.5/3.79	69.9/4.04, 3.93
C	6-β-Gal_bb_	104.2/4.70	71.3/3.53	73.4/3.65	69.0/3.96	73.5/3.79	70.0/4.04, 3.93
C	3,6-β-Gal_bb_	104.2/4.70	70.2/3.66	83.0/3.88	69.0/4.21	73.5/3.79	69.9/4.04, 3.93
C	3,6-β-Gal_bb_	104.2/4.70	70.2/3.65	83.0/3.90	69.0/4.21	73.5/3.79	69.9/4.04, 3.93
C	t-β-Gal_bb_	104.2/4.70	71.3/3.53	73.4/3.65	69.0/3.87	75.5/3.69	61.2/3.78
Hyp	set 1		60.4/4.33	35.4/2.62, 2.20	78.6/4.78	52.5/3.67, 3.57	
	set 2		60.1/4.22	35.0/2.54, 2.45	77.9/4.69	52.5/3.78,3.48	

## Data Availability

Data will be made available upon request.
